# Supplementary material for: Development of a High-Resolution Acoustic Sensor Based on ZnO Film Deposited by the RF Magnetron Sputtering Method
Source: Materials (Basel). 2021 Nov 14;14(22):6870. doi: 10.3390/ma14226870 (PMC8624261; doi:10.3390/ma14226870)
Supplement: Supplementary file 1 [file materials-14-06870-s001.zip › materials-1428554-supplementary.pdf]

Supplementary Materials

E-beam evaporation is a typical vacuum thin-film deposition system of the physical vapor deposition (PVD) method, which is a thin-film fabrication method using physical methods, and its schematic diagram appears in Figure S1 [39].

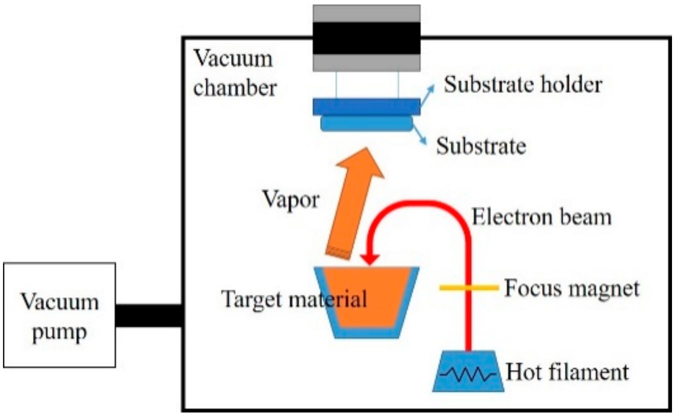

Figure S1. Schematic of the E-beam evaporation system.

The RF magnetron sputtering method was used for the ZnO thin-film deposition, the most fundamental part of the acoustic sensor. A schematic of the RF magnetron sputtering equipment is shown in Figure S2 [40]. In Figure S2, MFC (mass flow controller) is a controller to precisely control the amount of injected gas. The unit is sccm (standard cubic centimeter per minute).

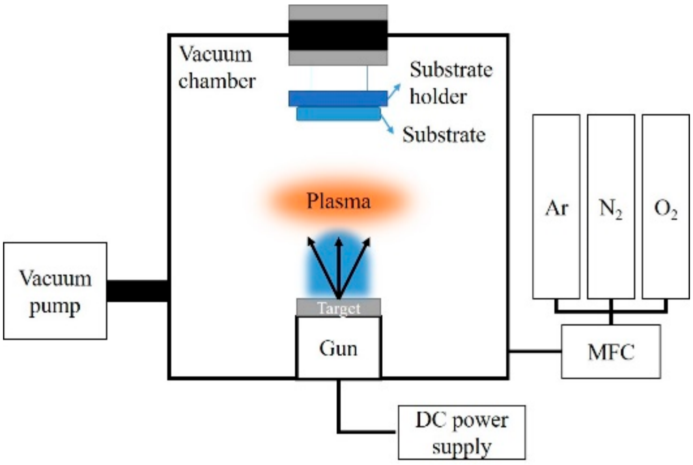

Figure S2. Schematic of the RF magnetron sputtering system.
